# Perianal mantle cell lymphoma mimicking an external thrombosed hemorrhoid: a case report

**DOI:** 10.1186/1752-1947-8-40

**Published:** 2014-02-05

**Authors:** Baris Gulcu, Ali Ozer, Hulya Ozturk Nazlioglu, Ersin Ozturk, Tuncay Yilmazlar

**Affiliations:** 1 Department of General Surgery, Uludag University Faculty of Medicine, Görükle 16059, Bursa, Turkey; 2 Department of Pathology, Uludag University Faculty of Medicine, Görükle 16059, Bursa, Turkey

**Keywords:** Inguinal lymph nodes, Mantle cell lymphoma, Perianal mass

## Abstract

**Introduction:**

Malignancies of the anal margin and perianal skin are relatively uncommon, and account for approximately 2% to 3% of all anorectal malignancies. Despite the fact that gastrointestinal presentation of lymphoma is not uncommon, primary localization of mantle cell lymphoma to the perianal region is rare.

**Case presentation:**

We present the case of a 64-year-old Turkish man with a rapidly progressive perianal mass. Our patient had previously required medical treatment on multiple occasions for hemorrhoidal disease; however, the treatment was ineffective and gross lymph nodes were noted in his left inguinal region. Following excision of the mass and his lymph nodes, the pathological diagnosis of both tissues was mantle cell lymphoma.

**Conclusion:**

Although gastrointestinal presentation of non-Hodgkin lymphoma is common, the literature includes only a few cases of perianal localization. Our case illustrates the importance of suspicion and complete examination of perianal masses. In practice, examination of the inguinal region should be a part of routine proctological examination.

## Introduction

Malignancies of the anal margin and perianal skin are relatively uncommon, and account for approximately 2% to 3% of all anorectal malignancies. The most common anorectal malignancies are squamous cell carcinoma, Bowen’s disease (high-grade intraepithelial squamous cell carcinoma), adenocarcinoma, basal cell carcinoma and malignant melanoma. A Buschke-Lowenstein tumor, or verrucous carcinoma, is a benign lesion of the perianal margin and is not considered an anorectal malignancy [1].

Mantle cell lymphoma (MCL) is a subtype of B-cell non-Hodgkin lymphoma and comprises about 7% of all cases of adult non-Hodgkin lymphoma. Approximately 25% of patients with MCL present with extra-nodal disease, of which 20% occur in the gastrointestinal (GI) tract. Multiple lymphomatous polyposis is a rare primary GI manifestation of MCL [2]. The literature includes few reports of MCL localized to the perianal region, and we therefore present the case of a patient with a perianal mass that mimicked hemorrhoidal disease but was diagnosed as MCL.

## Case presentation

**Figure 1:**
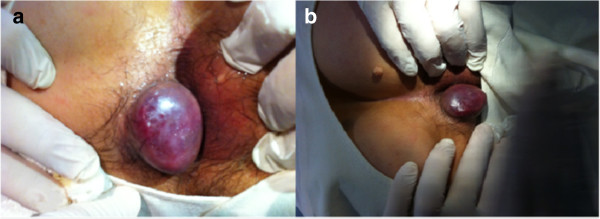
(a,b) Perianal mass mimicking thrombosed hemorrhoidal disease.

A 64-year-old Turkish man presented to our surgical clinic with a rapidly progressive perianal mass (Figure 1a,b). Our patient had previously required medical treatment on multiple occasions for hemorrhoidal disease, but the lesion increased to 4cm over the course of weeks despite medical treatment. The lesion was located in his perianal region and mimicked a thrombosed hemorrhoid, although it had a smooth and bright surface, and was painless during examination. Lymph nodes were observed in his left inguinal region, measuring approximately 2cm and 3cm in diameter. His anal canal was normal based on anoscopy and there were no significant laboratory findings. An ultrasound examination of his inguinal region showed conglomerate lymph nodes on the left side. The lesions were considered to be malignant and surgery involving wide excision of the lesion and sampling of his lymph nodes was scheduled.

**Figure 2:**
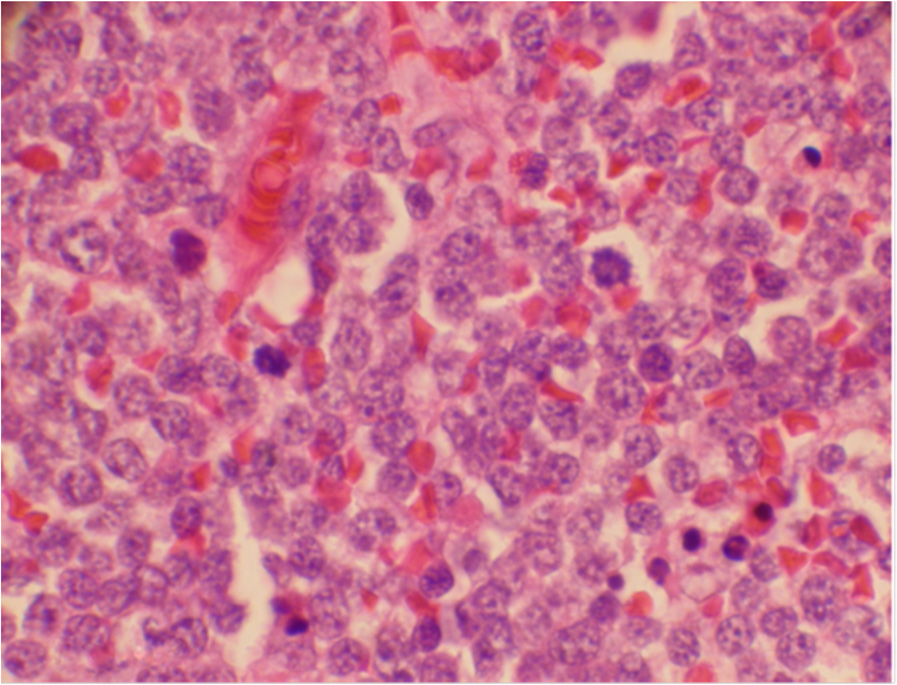
Diffuse infiltration of medium-sized neoplastic lymphoid cells.

Following excision of the mass and lymph nodes, a pathological diagnosis of MCL was made for both tissues (Figure 2). No evidence of systemic lymphoma was observed. Unfortunately, our patient received no additional treatment due to comorbidities that included severe cardiopulmonary insufficiency. He died as a result of pneumonia-associated sepsis 43 days after the surgery.

## Discussion

Patients with perianal lesions often present with common perianal complaints, including itching or burning, bleeding, pain, drainage or a mass [1]. However, the diagnosis and treatment of such lesions are often delayed due to the non-specific nature of symptoms. Lymphatic drainage of the anal canal is divided according to the location on the dentate line. Lymphatics affecting the anal canal that are proximal to the dentate line drain to the inferior mesenteric nodes. Lymphatics affecting the anal margin that are distal to the dentate line primarily drain to the inguinal nodes. Drainage of the perianal skin is also entirely to the inguinal nodes [1,3]; therefore, inguinal lymph node examination is a must when a perianal mass is detected.

MCL is an aggressive B-cell lymphoma with a propensity to involve extra-nodal sites, including the colon. Secondary colonic involvement of MCL occurs in 88% of patients. Primary GI lymphomas are relatively uncommon, representing 1% to 4% of malignant tumors of the GI tract [3]. Endoscopy reports indicate that localization of lymphoma within the GI is as follows: esophagus, 6%; stomach, 74%; duodenum, 34%; ileum, 48%; cecum, 14%; colon, 57%; rectum, 48%. Intestinal lesions of MCL present as multiple lesions in nearly 80% of patients, whereas a protruding mass occurs in 18% [4]. Only 26% of patients with MCL presented with GI symptoms, yet MCL was detected via histological and immunohistochemical analysis in the lower GI tract in 88% of patients and in the upper GI tract in 43% [5].

## Conclusion

Perianal masses have a variable etiology and most are benign; however, perianal presentation of unusual disorders such as MCL should always be a consideration. Although colorectal presentation of MCL is rare, a high index of suspicion among surgeons can prevent delays in diagnosis and treatment.

## Consent

Written informed consent was obtained from the patient for publication of this case report and all accompanying images. A copy of the written consent is available for review by the Editor-in-Chief of this journal.

## Abbreviations

GI: gastrointestinal; MCL: mantle cell lymphoma.

## Competing interests

The authors declare that they have no competing interests.

## Authors’ contributions

BG analyzed and interpreted the patient data regarding characteristics of the disorder. BG and AO were the major contributors in writing the manuscript. EO was the primary surgeon and had the main role in literature researching. HON was responsible for the pathological review. TY was the mentor in all processes and determined the pattern of case presentation. All authors read and approved the final manuscript.
